# Recombinant Marek’s disease virus expressing VP1 and VP2 proteins provides robust immune protection against chicken infectious anemia virus

**DOI:** 10.3389/fmicb.2024.1515415

**Published:** 2025-01-06

**Authors:** Chengfei Ge, Hangqiong Lu, Jinze Han, Guorong Sun, Shihao Li, Xingge Lan, Yongzhen Liu, Mengmeng Yu, Xinyun Hu, Mingxue Hu, Xiaole Qi, Hongyu Cui, Yulu Duan, Suyan Wang, Yuntong Chen, Xiaomei Wang, Yanping Zhang, Yulong Gao, Changjun Liu

**Affiliations:** ^1^Avian Immunosuppressive Diseases Division, State Key Laboratory for Animal Disease Control and Prevention, Harbin Veterinary Research Institute, Chinese Academy of Agricultural Sciences, Harbin, China; ^2^Jiangsu Co-Innovation Center for the Prevention and Control of Important Animal Infectious Disease and Zoonosis, Yangzhou University, Yangzhou, China

**Keywords:** chicken infectious anemia virus, Marek’s disease virus, CRISPR/Cas9, VP1, VP2, vaccine

## Abstract

Chicken infectious anemia (CIA) is a highly contagious disease caused by the chicken infectious anemia virus (CIAV), and it poses a serious threat to the poultry industry. However, effective control measures and strategies have not been identified. In this study, a recombinant Marek’s disease virus (rMDV) expressing the VP1 and VP2 proteins of CIAV was successfully constructed using CRISPR/Cas9, and a commercial Marek’s disease virus (MDV) vaccine strain was used as the vector. VP1 and VP2 expression by rMDV was confirmed by immunofluorescence assay and western blot analysis, which revealed robust *in vitro* expression. Further analysis showed that the VP1 and VP2 genes integrated into the MDV genome did not alter the growth kinetics of the virus and remained stable even after 20 passages, indicating the genetic stability of the recombinant virus. In animal studies, vaccination of one-day-old specific-pathogen-free chickens with rMDV induced high levels of CIAV-specific antibodies (1 × 10^5^) and neutralizing antibodies (1:2^5^) and a potent cellular immune response. Moreover, rMDV vaccination conferred an 85% protective index against challenge with a highly virulent strain of CIAV, significantly reducing the occurrence of anemia and thymic atrophy caused by CIAV infection and dramatically suppressing CIAV replication in the thymus. Collectively, these results highlight the potential of rMDV as a vaccine candidate for preventing and controlling CIAV infection, thus offering a new avenue for mitigating the impact of CIA on the poultry industry.

## 1 Introduction

Chicken infectious anemia (CIA) is a globally distributed disease primarily characterized by immunosuppression, aplastic anemia, and systemic lymphoid tissue atrophy in chicks, and it is caused by the chicken infectious anemia virus (CIAV) (McNulty, 1991). The virus is prevalent in areas with intensive poultry farming and spreads vertically from hens to chicks via eggs and horizontally through direct contact or contaminated environments. Chicks lacking maternal-derived antibodies (MDAs) or acquired antibodies to CIAV are primarily affected, resulting in clinical symptoms and even mortality (Jørgensen et al., 1995). CIAV infection in older chickens often leads to subclinical immunosuppression, increasing susceptibility to co-infections or secondary infections associated with other common pathogens, such as Marek’s disease virus (MDV) (Zhang et al., 2017a), Newcastle disease virus (NDV) (Su et al., 2018), and fowl adenovirus serotype 4 (FAdV4) (Yu et al., 2019), which manifest in more severe disease onset, further exacerbating the immune suppression and mortality in chicken flocks and leading to significant economic losses to the poultry industry (Vaziry et al., 2011).

CIAV is the sole member of the *Anelloviridae* family, and its genome consists of three partially overlapping open reading frames that encode the VP1, VP2, and VP3 proteins (Noteborn et al., 1991). The VP1 protein is the primary structural capsid protein containing numerous neutralizing antigenic epitopes, and it serves as the main immunogenic protein of CIAV (Renshaw et al., 1996). The VP2 protein serves as a scaffold protein that aids in the correct folding of VP1 (Koch et al., 1995). The simultaneous synthesis of both proteins results in the formation of a specific spatial conformation that exposes the essential antigenic epitopes for neutralization, thereby stimulating the host to produce CIAV-specific neutralizing antibodies (Noteborn et al., 1998).

Recombinant live vaccines use viruses or bacteria as carriers to express exogenous genes inserted via genetic engineering technology (Ewer et al., 2016). MDV, which belongs to the *Herpesviridae* family, possesses several advantageous properties that make it an attractive vector for vaccine development (Wang et al., 2024). Compared to other viral vectors, MDV has a large double-stranded DNA genome containing numerous non-essential regions for replication, which enables the insertion of multiple foreign genes (Kamel and El-Sayed, 2019). In addition, live MDV vaccines can establish lifelong persistent infections in the host, thus providing prolonged antigen exposure and sustained immune stimulation, which are beneficial for maintaining long-term immunity without the need for booster vaccinations (Venugopal, 2000). In addition, Marek’s disease (MD) is primarily controlled by vaccination administered in *ovo* or via subcutaneous injection on the hatch day (Williams and Hopkins, 2011). The poultry industry has well-established protocols and infrastructure for MDV vaccination, making it cost-effective to adapt them to recombinant MDV-based vaccines (Hein et al., 2021). Significant progress has been made in the use of MDV as a vaccine vector to develop recombinant vaccines that protect against various poultry diseases, including infectious bursal disease (IBD) (Li et al., 2016), Avian Leukosis Virus Subgroup J (ALV-J) (Liu et al., 2016), and reticuloendotheliosis virus (REV) (Li et al., 2020).

Currently available vaccines for CIAV infection are live whole virus vaccines that have been continuously attenuated in cell lines or chicken embryos (Li K. et al., 2017; Li Y. et al., 2017). However, the primary concern with current CIAV vaccines lies in safety, primarily offering passive immunity, which limits their effectiveness in embryonic and neonatal immunization. Therefore, this study developed a simple, safe, and effective vaccine strain suitable for early immunization by inserting the VP1 and VP2 genes from a field CIAV strain into a licensed MDV vaccine strain (rMS-∆Meq) (Zhang et al., 2017b) using CRISPR/Cas9 gene editing technology. Vaccination of one-day-old specific-pathogen-free (SPF) chicks with this novel vaccine not only initiates an earlier immune response but also has the potential to provide active immunity, thereby conferring efficient protection against CIAV infection. As a result, the proposed recombinant virus presents itself as a promising candidate for a vaccine strain.

## 2 Materials and methods

### 2.1 Chickens

One-day-old SPF chicks were obtained from Boehringer-Ingelheim Biotechnology Co. Ltd. (Beijing, China), hosted on site at the Animal Facility of Harbin Veterinary Research Institute (HVRI), and maintained at the highest standard of animal care under SPF conditions. The experimental chickens were anesthetized through CO_2_ inhalation anesthesia, followed by exsanguination, and subsequent dissection and sampling. All animal experiments were approved by the Animal Ethics Committee of the HVRI and conducted in accordance with the authorized protocol (No. 230807-01-GR).

### 2.2 Cells

DF-1 and MSB-1 cells were cultured in Dulbecco’s modified Eagle’s medium (DMEM; Catalog No. D6429, Gibco) supplemented with 10% fetal bovine serum (FBS, catalog No. 10099-141, Gibco) and antibiotics (100 U/mL penicillin and 100 μg/mL streptomycin, InvivoGen), at 37°C and 5% CO_2_ in a humidified incubator.

### 2.3 Viruses

MDV vaccine strain rMS-∆Meq was used as the parental virus to produce recombinant MDV. All MDV stocks were propagated in CEFs derived from 9-day-old SPF chicken embryos. CIAV JL17P10 was used as the challenge strain, and the HeN/193001 strain (Li et al., 2021) was used as the parental virus to amplify VP1 and VP2.

### 2.4 Plasmids

The pX330, pCAGGS, pCDNA3.1, and pEGFP-N1 vectors were purchased from Addgene (United States). Recombinant plasmids pCAGGS-VP1 and pCDNA3.1-VP2 capable of expressing VP1 or VP2 proteins were amplified from the HeN/193001 strain. pCAGGS and pCDNA3.1 vectors were digested with BglII (catalog No. R0144V, NEB) or EcoRI (catalog No. R3101S, NEB), followed by purification using a QIAquick Gel Extraction Kit (catalog No. 28704, OMEGA) according to the operation manual. Finally, VP1 and VP2 fragments were sequentially cloned into the pCAGGS and pCDNA3.1 vectors using the In-Fusion cloning kit (catalog No. C112-01, Vazyme).

Two sgRNA sequences targeting the UL41 region of rMS-∆Meq genome were designed using an online platform (CRISPR Guide RNA Design Tool | Benchling). Primers UL41-sgRNA1-F/R and UL41-sgRNA2-F/R for cloning sgRNAs were synthesized by Ruibiotech (Harbin, China). The CRISPR-Cas9 vector pX330 was digested with BbsI-HF (catalog No. R3539S, NEB) and purified using a QIAquick Gel Extraction Kit. The two sgRNAs were then cloned into linearized pX330 using T4 DNA ligation (catalog No. N103-01, Vazyme). To generate the donor plasmid, the homologous arms upstream and downstream of the Cas9 cutting site within UL41 were amplified using primers LHR-F/R and RHR-F/R, the VP1-P2A-VP2 ORF was amplified using primers VP1-JF1/R1 and VP2-JF1/R1, and the eGFP reporter cassette was amplified using primers eGFP cassette-kF/kR. All fragments were sequentially cloned into the pCAGGS vector using the In-Fusion cloning kit. The primers used in this study are listed in Table 1.

**Table 1:** Primers used in this study.

Primer name	Primer sequence(5′-3′)
VP1-F1	GCTGTCTCATCATTTTGGCAAAGAATTCGCCACCATGGCCCGCCGCGCCCGCCGCCCACGCGGCCGCTTC
VP1-R1	TTTTTGGCAGAGGGAAAAAGATCTTGGTCCTGGATTTTCTTCCACGTCTCCTGCCTGCTTCAACAATGAGAAGTTAGTTGCGGGCTGGGTGCCCCAGTACA
VP2-F1	GAAGAAAATCCAGGACCAATGCACGGAAATGGCGGA
VP2-R1	GCAGAGGGAAAAAGATCTTCACACAATTCTCACTGGAGCA
GFP cassette-kF	TCCCTCGACCTGCAGCCCAAGCTTCGTTACATAACTTACGGT
GFP cassette-kR	TATGACCATGATTACGCCAAGCTTTAAGATACATTGATGAGTTT
LHR-F	ACATTTCCCCGAAAAGTGCCACCTGGTCTAGGACACCTTCGAGCGTTGAG
LHR-R	TAACTAGTCAATAATCAATGTCTACCGGAGGTACGCCCTCTTAA
RHR-F	AAACTCATCAATGTATCTTACAGTGTAATTATCGAATCGTCG
RHR-R	CAGCTATGACCATGATTACGCCATGGGTTCTTCACGCAACCTAC
UL41-sgRNA1-F	CACCGTTTATAAAACGTATACCGG
UL41-sgRNA1-R	AAACCCGGTATACGTTTTATAAAC
UL41-sgRNA2-F	CACCGTACGGATGTTGGAGAGGCG
UL41-sgRNA2-R	AAACCGCCTCTCCAACATCCGTAC
VP1-JF1	CTTCCGCAAGGCCTTCCA
VP1-JR1	CAGTACATGGTGCTGTTG
VP2-JF1	AAATGGCGGACAGCCAGCT
VP2-JR1	GGGGTAGTAAATGGTCTT
UL41-JF	GTAGCAATGACATGCTTA
UL41-JR	GAAAGGTTTGAAACCCCG
rMS-F	GGGAGAAGGCGGGCAGTCGA
rMS-R	GGAGGTTGGGAACCGGAGCA
rMS-probe	FAM-ACTCCTCCACCTCCCTCACCGGATGAAC-TAMRA
CIAV-F	AATTTCGACATCGGAGGAG
CIAV-R	GGAAGCGGATAGTCATAGTAGAT
CIAV-probe	FAM-AGCGGTATCGTAGACGAGCTTTTAGGAAGGC-TAMRA
OVO-F	CACTGCCACTGGGCTCTGT
OVO-R	GCAATGGCAATAAACCTCCAA
OVO-probe	FAM-AGCGGTATCGTAGACGAGCTTTTAGGAAGGC-MGB

The correctness of all plasmid constructions was confirmed through Sanger sequencing.

### 2.5 Generation of rMDV delivering CIAV VP1 and VP2

To generate the recombinant virus expressing VP1 and VP2 proteins, CEF cells grown in 6-well plates were co-transfected with 0.5 μg of CRISPR-Cas9 plasmids and 1 μg of donor plasmid using TransIT-X2^®^ Dynamic Delivery System (catalog No. MIR6000, Mirus Bio) according to per the manufacturer’s instructions. At 12 h post-transfection (h.p.t.), the cells were then infected with rMS△Meq at 100 plaque-forming unit (PFU). Approximately 4 days post-transfection, individual cells expressing eGFP were sorted into 96-well plates using fluorescence-activated cell sorting (FACS) with a SONY-MA900 Flow Cell Sorter (SONY, JAPAN). Subsequently, the obtained rMDVs were purified through multiple rounds of plaque isolation. The presence of the parental virus was excluded using the primers UL41-JF/JR, while the insertion of foreign genes was confirmed through PCR amplification using VP1-JF1/R1 and VP2-F/R primers, followed by Sanger sequencing.

### 2.6 IFA

CEF cells were cultured in 6-well plates and infected with either the parental virus or rMDV at a dose of 100 PFU. At 72 h post-infection (h.p.i.), the infected cells underwent three washes with phosphate-buffered saline (PBS), followed by fixation with 4% paraformaldehyde for 20 min and permeabilized with 0.1% TRITON^®^ X-100 (catalog No. 648463, Merck) in PBS for 15 min at 25°C. Subsequently, the CEFs were incubated with an anti-VP1/2 monoclonal antibody (Mab; produced and preserved in our laboratory) as the primary antibody in PBS for 2 h at 37°C. Recombinant plasmids pCAGGS-VP1 and pCDNA3.1-VP2 served as positive controls for detecting VP1/2 protein expression. Following another three washes with PBS, CEFs were incubated with rabbit anti-mouse IgG H&L Alexa Fluor^®^ 594 (1:100) (catalog No. ab150116; Abcam) in PBS for 45 min at 37°C. The results were observed by an Axio fluorescence microscope (Carl Zeiss, Germany).

### 2.7 Western blot

CEF cells were cultured in 6-well plates and infected with either the parental virus or rMDV at a dose of 100 PFU. MDCC-MSB1 cells were infected with the CIAV HeN/193001 strain at an MOI of 0.01. At 72 h.p.i., the infected cells underwent three washes with PBS. The supernatants collected from the infected cells were lysed using NP-40 lysis buffer (Beyotime) and then subjected to 12% SDS-PAGE. For western blot analysis, anti-VP1/2 monoclonal antibody was used as the primary antibody, followed by DyLight 800 (catalog No. 926-32210, LiCor Bio-Sciences) as the secondary antibody. Concurrently, beta-actin protein levels in the cell lysates were detected using a mouse monoclonal antibody to beta-actin (catalog No. A1978, Sigma) at a dilution of 1:1,000 as the primary antibody, and a goat polyclonal anti-mouse IgG antibody conjugated to HRP (catalog No. A00160, GenScript Laboratories) at a dilution of 1:1000 as the secondary antibody. Protein expression was visualized using a CCD Azure c600 imaging system (Azure Biosystems, Inc., United States).

### 2.8 Stability and growth property assay

To investigate the growth properties of recombinant MDV, cells were cultured in 6-well plates and inoculated with 100 PFU of recombinant and parental viruses. The cells were then harvested every 24 h, and serial dilutions were inoculated into CEF cells seeded in 6-well plates. Plaques from different dilutions were counted 5 days later. The experiment was independently repeated three times. To evaluate the genetic stability of the recombinant virus, it was passaged 20 times in CEF cells. The inserted VP1 and VP2 genes were detected by PCR using primers VP1-JF1/R1 and VP2-F1/R1, followed by sequencing. Viral DNA was extracted from infected CEF cells every 3rd to 4th passage. Expression of the VP1 and VP2 proteins was confirmed after 20 passages through fluorescence assays, as described above. The experiment was independently repeated three times.

### 2.9 Animal experiments

A total of 55 1-day-old SPF chicks were randomly divided into four groups. Thirty chickens were vaccinated subcutaneously with 5,000 PFU of rMDV on day 1. At 3 weeks post-vaccination, 15 chickens were challenged with 10^5.5^ TCID_50_ of the virulent CIAV strain JL17P10, and the remaining 15 served as the vaccination-only group. The CIAV challenge control group consisted of 15 chickens subcutaneously injected with 10^5.5^ TCID_50_ of the virulent CIAV strain JL17P10 on day 21. The healthy control group consisted of 10 chickens injected with the corresponding solvent during the experimental period (mock treatment).

Peripheral blood mononuclear cells (PBMCs) from chickens in the immune and healthy control groups were isolated using a chicken PBMC isolation kit (catalog No. LTS1090C TBD) according to the manufacturer’s instructions. Cell-Mediated immune (CMI) responses in PBMCs were evaluated at 3–5 weeks post-vaccination (w.p.v.) by stimulation with ConA (concanavalin A) and LPS (lipopolysaccharide), as previously described (Jones, 2019; Raulf, 2019), and gene expression analysis of IFN-*γ* was conducted using an ELISA kit (catalog No. MM-0520O1, Meimian) at 4–5 w.p.v., according to the manufacturer’s instructions.

In the first week post-challenge (w.p.c.), blood samples were collected from each group and hematocrit (Hct) was measured using capillary tubes following the standard procedure (Wani et al., 2016). Additionally, thymus atrophy in chickens was evaluated using the thymus-to-body weight index (TBIX) and thymus index during this period. The thymus index was calculated as follows: $\frac{X\;\mathrm{CIAV challenged chicken}\left(\;\mathrm{TBIX}\right)}{\bar{x\;}\;\mathrm{healthy control group}\;\left(\mathrm{TBIX}\right)}$ . The thymus was considered atrophied when the thymus index value ≤0.8 . All chickens were dissected 7 and 12 days after the challenge, and the clinical symptoms of each chicken were recorded.

### 2.10 Expression analysis *in vivo*

To assess the expression of VP1 protein in recombinant MDV *in vivo*, thymus, liver, and spleen tissues were collected from randomly selected vaccinated chickens at 14 and 42 days post-vaccination (d.p.v.). The tissues were treated with RIPA Lysis Buffer (catalog No. K1020, APEXBIO), and the extracted proteins were subjected to 12% SDS-PAGE. Western blot analysis was performed using an anti-VP1 monoclonal antibody as the primary antibody and DyLight 800 as the secondary antibody. Protein expression was visualized using a CCD camera Azure c600 imaging system.

### 2.11 ELISA and neutralizing antibody titer

Serum samples were collected weekly from immunized and healthy control chickens throughout the experimental period. To evaluate the level of enzyme-linked immunosorbent assay (ELISA) antibody titers induced by rMDV, an ELISA was conducted to determine the antibody status and antibody titers against CIAV using the Chicken Infectious Anemia Virus Antibody Test Kit (catalog No. 9908702, IDEXX) at 1–6 w.p.v. According to the manufacturer’s instruction, the serum samples with the ELISA S/N ≤ 0.6 and antibody titer ≥2050 were considered positive. To assess the neutralizing antibody titer produced by rMDV, sera collected at 6 w.p.v. were analyzed for neutralizing activity against CIAV infection in MDCC-MSB1 cells based on a previously described method (Moeini et al., 2011a).

### 2.12 Flow cytometry analysis

At 3–5 w.p.v., PBMCs collected from the immune and control groups of chickens were analyzed using flow cytometry (BD Biosciences, United States) to quantify the expression levels of CD4+ and CD8+ T lymphocytes. PBMCs were incubated for 30 min at 4°C with 2 μg each of anti-chicken CD3-SPRD, CD45-APC, CD4-FITC and CD8-PE antibodies (Southern Biotech, United States) diluted in PBS containing 5% FBS. Subsequently, the relative content of each T-cell subset was estimated by analyzing the fluorescence intensity of the cells using a flow cytometer (A60 Universal, Apogee Flow Systems, UK).

### 2.13 qPCR

To assess the replication ability of rMDV in chickens, the thymuses of randomly selected vaccinated chickens were collected at 14, 21, 28, and 35 d.p.v. and quantified by absolute quantitative real-time PCR using Premix Ex Taq (catalog No. R390B, TaKaRa). DNA was extracted from the thymus using a Tissue DNA Isolation Mini Kit (catalog No. DC102-01, Vazyme). MDV genome copies per 1 × 10^6^ cells were quantified using real-time PCR. At 12 days post-challenge (d.p.c.), thymuses were collected from each group and viral loads were quantified by absolute quantitative real-time PCR using Premix Ex Taq. CIAV genome copies per 1 × 10^4^ cells were quantified using real-time PCR. All the primers utilized are detailed in Table 1, and viral loads were determined using an equation derived from the standard curve.

### 2.14 Histopathological analysis

At 7 d.p.c., chicken thymuses were collected, fixed in 10% neutral buffered formalin, fixed with paraformaldehyde, and stained with hematoxylin and eosin (H&E staining). Pathological changes were examined using Image Pro Plus software (version 6.0; Media Cybernetics Inc., United States), which can measure staining.

### 2.15 Statistical analysis

One-way analysis of variance (ANOVA) of GraphPad Prism software (version 10.2.0 for Mac, United States) was used to evaluate intergroup differences. Differences were considered significant at *p < 0.05, **p < 0.01, ***p < 0.001, and ****p < 0.0001.

## 3 Results

### 3.1 Successful rescue of rMDV expressing CIAV VP1 and VP2 proteins

**Figure 1:**
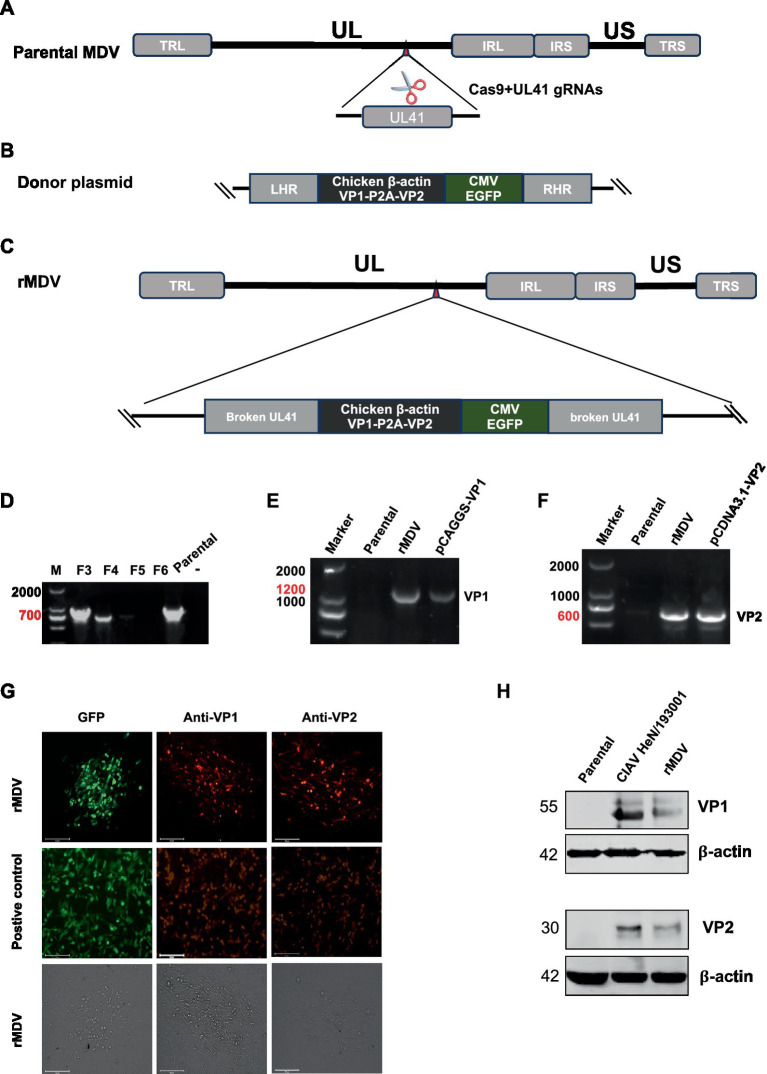
Strategy for the construction and generation of recombinant virus (rMDV). **(A–C)** Strategy for the construction of recombinant virus. **(D)** Identification of purification of recombinant virus. Cells from isolated plaque were lysed, and then the recombinant virus was isolated through five rounds of plaque isolation by FACS and identified via PCR using primers located at the flanking region of the insertion site. The bands (700 bp) amplified from passaged recombinant virus-infected cells indicate the presence of the parental virus. None of the amplified bands indicating the pure recombinant strain were obtained by clone screening to the 5th generation. **(E,F)** PCR analysis of viral genome isolated from infected CEF cells using specific primers to identify the VP1 and VP2 gene. VP1: 1,200 bp; VP2: 600 bp. DNA Marker: 2,000 bp. **(G)** Confirmation of rMDV expressing the VP1 and VP2 proteins by indirect immunofluorescence assay. CEF cells were infected with the recombinant virus at 100 PFU. Then, the CEF cells were harvested at 72 h.p.i. and expression of the genes was determined by IFA. The plasmids pEGFP-N1, pCAGGS-VP1 and pCDNA3.1-VP2 were transfected into DF-1 cells used as positive controls, respectively. Scale length, 125 μm. **(H)** Detection of VP1 and VP2 expression from rMDV via western blot using anti-VP1/2. CEF cells were infected with the recombinant virus at 100 PFU. Then, the CEF cells were harvested at 72 h.p.i. and processed to prepare cell lysates. CIAV HeN/193001 was detected as the positive control. rMS∆Meq was detected as the negative control. The VP1 and VP2 proteins of CIAV were located at approximately 55 KDa and 30 KDa, respectively.

To construct recombinant MDV expressing the VP1 and VP2 proteins from CIAV using CRISPR/Cas9 gene editing technology, we constructed two p-sgRNAs (Figure 1A) targeting the UL41 region of the rMS- Δ Meq genome and one donor plasmid containing CIAV VP1 and VP2 ORFs linked by a P2A sequence, along with an eGFP expression cassette (Figure 1B). To rescue the recombinant virus, two CRISPR plasmids and the donor plasmid were co-transfected into CEF cells for 12 h. We then infected the transfected-cells with parental MDV (rMS-△Meq vaccine strain), the single cell clones expressing eGFP fluorescence were sorted by FACS at 4 days post-infection (d.p.i.). After adequate proliferation, these single-cell clones were inoculated into CEFs for plaque purification. The recombinant virus obtained from the 5th round of plaque purification no longer contained sequences specific to the parental strain. PCR results revealed CIAV VP1 and VP2 gene sequences, indicating the successful acquisition of the desired recombinant virus. This strain was designated as rMDV-VP1/VP2-eGFP (rMDV) (Figures 1C–F). According to the immunofluorescence assay (IFA) results, VP1 and VP2 protein expression was indicated by red fluorescence in the infected cells while eGFP expression was indicated by green fluorescence (Figure 1G). Similarly, western blot results further confirmed the expression of the VP1 protein (∼55 kDa) and VP2 protein (∼30 kDa) in rMDV-infected cells (Figure 1H). These results indicate that rMDV-expressing CIAV antigen proteins VP1 and VP2 were successfully rescued.

### 3.2 rMDV exhibited reliable growth properties and genetic stability

**Figure 2:**
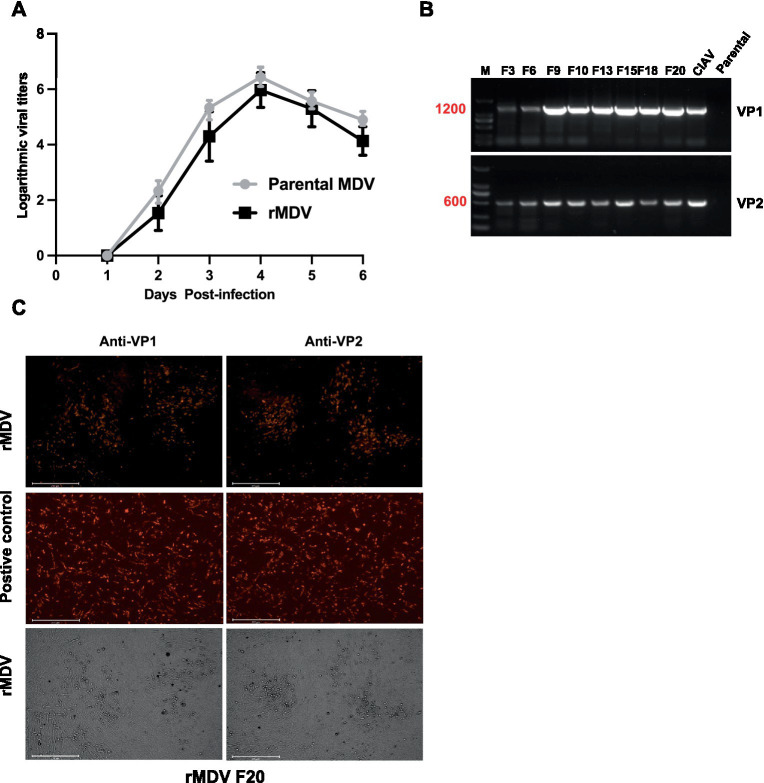
Characterization of the recombinant MDV virus (rMDV). **(A)** Comparison of the replication kinetics of the recombinant virus. Data are presented as the means ± standard deviations (S.D.) from three independent experiments. Statistical significance was set at *p* < 0.05. **(B)** VP1 and VP2 genes from rMDV passaged 20 times in CEFs were amplified by PCR. M: 2,000 DNA Marker. **(C)** VP1 and VP2 expression from rMDV passaged 20 times in CEFs with indirect immunofluorescence assay. The plasmids pCAGGS-VP1 and pCDNA3.1-VP2 were transfected into DF-1 cells used as positive controls, respectively. Scale bar, 125 μm, respectively.

To further characterize rMDV, we assessed its replication ability. The growth kinetics results indicated that its replication ability was not reduced and did not show a significant difference compared to that of the parental strain, suggesting that inserting the VP1 and VP2 genes into the UL41 region did not affect the replication of rMDV (Figure 2A). Next, we evaluated the genetic stability of rMDV through 20 rounds of sequential passaging in primary CEFs. The PCR results showed that the expected sequence sizes for the VP1 and VP2 genes were successfully amplified from rMDV (Figure 2B). The IFA results further revealed VP1 and VP2 protein expression in rMDV via red fluorescence, even after the 20th generation (Figure 2C). These results revealed that rMDV exhibited reliable growth properties and genetic stability.

### 3.3 Replication ability and expression of exogenous protein of rMDV *in vivo*

**Figure 3:**
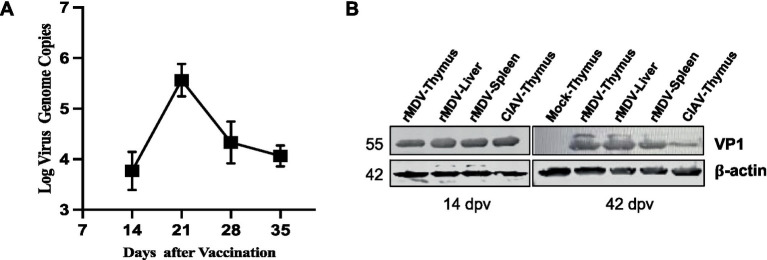
Replication and exogenous protein expression of rMDV *in vivo*. **(A)** rMDV replication kinetics in the thymus after vaccination according to RT-PCR analysis *in vivo*. Data are presented as the means ± standard deviations (S.D.) from three independent experiments. Statistical significance was set at *p* < 0.05. **(B)** *In vivo* expression analysis of the viral VP1 protein in the thymus, liver, and spleen by western blot at 14 and 42 days post-vaccination. CIAV HeN/193001 was detected as the positive control. rMS∆Meq was detected as the negative control. The VP1 protein of CIAV was located at approximately 55 KDa.

To assess the replication ability and expression of exogenous proteins of rMDV in chickens, we examined its replication kinetics in the thymus and expression of exogenous proteins in immune organs. The RT-PCR results revealed that viral titers gradually increased after immunization, peaking at 21 d.p.v. and displaying robust replication ability in chickens (Figure 3A). Western blot results confirmed the expression of the VP1 protein (~55 kDa) of rMDV in the thymus, liver, and spleen (Figure 3B). These findings indicated that rMDV exhibits robust replication capabilities and effectively expresses exogenous proteins *in vivo*.

### 3.4 rMDV induced humoral immunity in chickens

**Figure 4:**
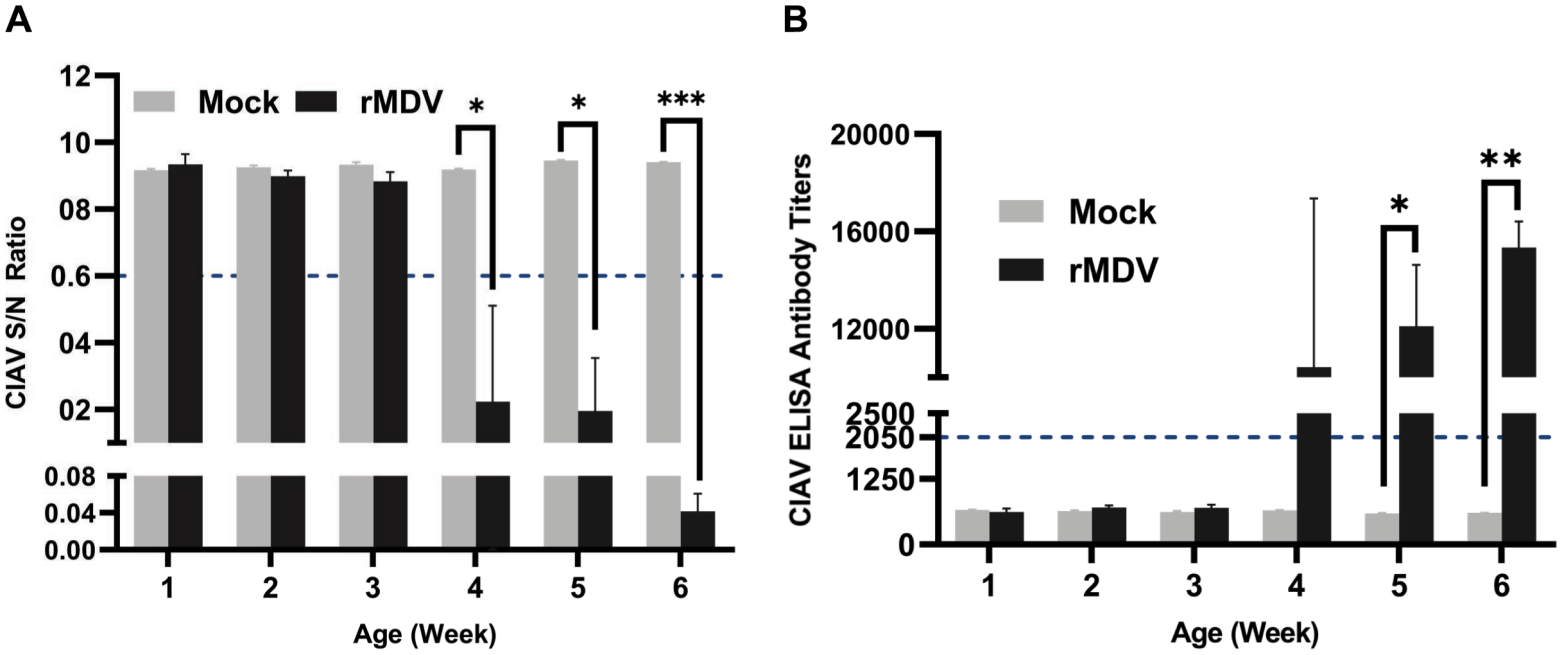
CIAV specific serum antibody response in the experimental chicken. Serum samples were collected weekly and detected using a commercial CIAV Antibody Test Kit for competitive ELISA. **(A)** Detection the S/N value of CIAV antibody in immunized animals during 42 days post-vaccination. **(B)** Detection of CIAV antibodies titers against CIAV in immunized chickens during 42 days post-vaccination. The presence of antibody was determined as positive for samples with a (S/N) ratio ≤ 0.6 or antibody titers ≥2,050 for each sample. Data are presented as the means ± standard deviations (S.D.) from five birds in each group. Statistical significance was set at *p* < 0.05.

To assess the humoral immune response induced by rMDV, serum samples were collected weekly from immunized chickens for 1–6 w.p.v. ELISA results indicated 100% positivity rates at 4 w.p.v., which persisted throughout the study period (Figure 4A). Simultaneously, the antibody titers against ClAV surpassed 2,050 at 4 w.p.v. and continued to increase during the six-week observation period, peaking above 1 × 10^5^ (*p* < 0.0001) at 6 w.p.v. (Figure 4B). Furthermore, the number of specific neutralizing antibodies against CIAV in vaccinated chickens reached 2^5^ at 6 w.p.v. (Table 2). These data indicated that a single immunization of SPF chickens with rMDV can induce a potent humoral immune response against CIAV.

**Table 2:** Neutralization activities of the serum antibodies in the vaccinated groups at 6 weeks post-vaccination (w.p.v.).

Sera groups	No. of positive	VN antibody titer	VN status
rMDV	5/5	1:2^5^	Positive
Mock	0/5	1:2^0^	Negative
Anti-CIAV serum (positive control)	5/5	1:2^4^	Positive

### 3.5 rMDV enhanced cell-mediated immunity in chickens

**Figure 5:**
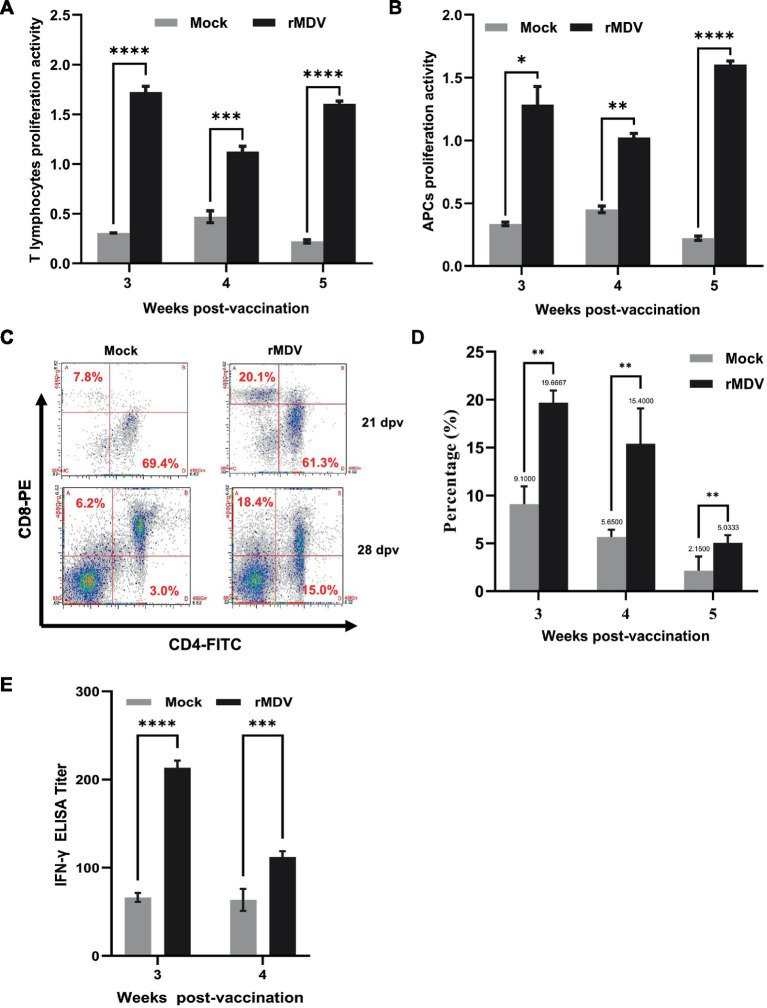
Assessment of cell-mediated immune (CMI) response in chicken after vaccination. Lymphocyte proliferation response in chicken at 3, 4, and 5 weeks after vaccination using **(A)** ConA- and **(B)** LPS-activated PBMCs. The PBMCs from the experimental birds (*n* = 5) were stimulated, and antigen-specific lymphocyte proliferation was expressed as the stimulation index (mean ± SE). Different superscripted capital letters indicate the time effect (*p* < 0.0001), while small superscripted letters indicate the treatment effect (*p* < 0.0001) in the vaccinated groups. **(C)** Flow cytometric analysis for CD4+ and CD8+ T-cell subsets in the blank group and vaccination group. **(D)** Percentage of CD8+ T cells in PBMCs at 3, 4, and 5 weeks after vaccination in birds in different groups. Bars (mean ± SE) indicate the representative data of a single experiment, and data with different small superscripted letters indicate the treatment effect (*p* < 0.0001). **(E)** Detection of IFN- γ antibody titers of immunized animals by ELISA at 3 and 4 weeks after vaccination. Data are presented as the means ± standard deviations (S.D.) from five birds in each group. Statistical significance was set at *p* < 0.0002.

To evaluate the cell-mediated immune response induced by rMDV, lymphocyte proliferation was first checked in PBMCs from both healthy control and rMDV-vaccinated chickens following stimulation by ConA and LPS. The results indicated that PBMCs from rMDV-immunized chickens exhibited significantly increased proliferative activity upon stimulation with both stimuli (Figures 5A,B). Additionally, we performed T-cell phenotyping analysis using flow cytometry, and the results showed that the proportion of CD8+ T cells in PBMCs from the rMDV-immunized group was significantly higher than that from the mock chickens (Figures 5C,D). Moreover, the concentration of IFN-*γ* in the serum samples was measured using ELISA, which indicated significantly higher IFN-γ levels in the immunized group than the control group (Figure 5E). These data indicate that vaccination of SPF chickens with a single dose of rMDV promoted cell-mediated immunity.

### 3.6 rMDV vaccination provided sufficient protection against CIA in chickens

**Figure 6:**
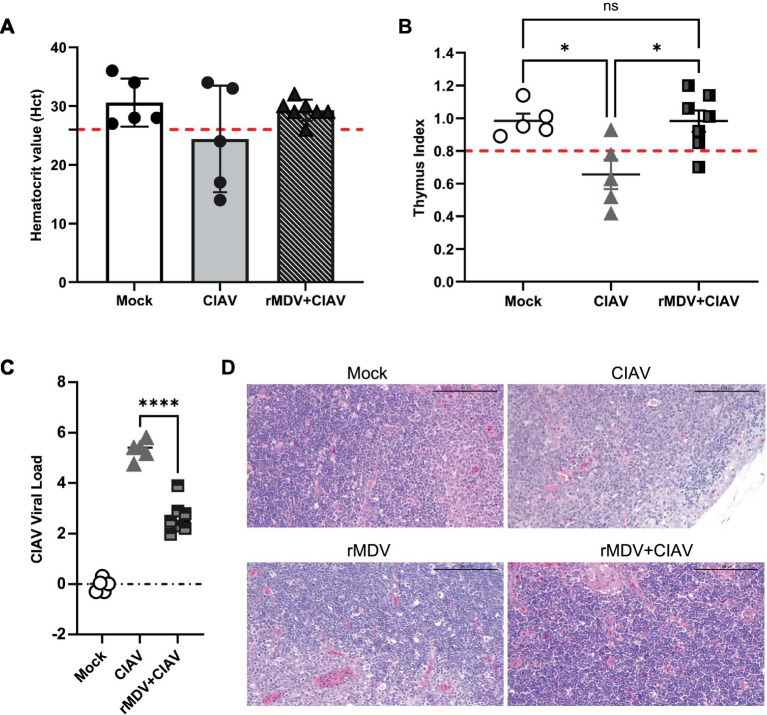
Protective efficacy of the recombinant vaccines against CIAV challenge. **(A)** Hematocrit value (Hct) after CIAV challenge. Hct < 27 indicates anemia. **(B)** Incidence rate of thymic atrophy evaluated by the thymus index scores for chickens after CIAV challenge. A score < 0.8 means thymic atrophy. **(C)** Determination of viral load in thymus at 12 days post-challenge. Data are presented as the means ± standard deviations (S.D.) from seven birds in of the vaccinated group and five birds in the blank control group and CIAV challenge group. Statistical significance was set at *p* < 0.05. **(D)** Representative images of the microscopic appearance of the thymus in chicks challenged with JL17P10. Chicks were vaccinated with rMDV (vaccinated chicks) or PBS (unvaccinated chicks) and then challenged intramuscularly with 1.5 mL of JL17P10 (5.5 × 10^5^ copy/mL). The thymuses were examined macroscopically at 7 days post-challenge.

To evaluate the anemia symptoms of CIAV infection, blood samples were collected using anticoagulants from each group 7 d.p.c. The Hct results revealed that Hct values dropped below 27 in 3 out of 5 chickens in the ClAV-challenged control group (*p* < 0.01), which was significantly higher than that in the vaccinated group (1 out of 7, *p* < 0.0001, Figure 6A), indicating that rMDV immunization nearly restored Hct levels to those of healthy control chickens. To evaluate the thymic atrophy symptoms of CIAV infection, the thymus:body index (TBIX) and thymus index were measured in each group at 7 d.p.c. The results indicated that vaccinated chickens exhibited significantly higher average TBlX values (4.15 ± 0.29, *p* < 0.0001, 4.50 + 0.48, *p* < 0.01) compared to that of the ClAV challenge control group (2.80 ± 0.39, *p* < 0.001, 4.46 + 0.85, *p* < 0.05, Table 3). Similarly, the thymus index results further confirmed that thymic atrophy occurred in 1 out of 7 members of the vaccinated group, which was significantly lower than that in the ClAV-challenged control group (4 out of 5, Figure 6B), suggesting that rMDV immunization prevented thymic atrophy. Correspondingly, to evaluate the viral load of CIAV infection, thymuses were collected from each group at 12 d.p.c. RT-PCR results showed that CIAV was detected in both the CIAV challenged control and vaccinated groups but not in the healthy control group. The CIAV load in the vaccinated group was 2.49 × 10^4^ copies/mL, whereas in the CIAV-challenged control group was 5.41 × 10^4^ copies/mL, indicating a lower level of viral load compared to the CIAV-challenged chickens (Figure 6C). Furthermore, histopathological examination results indicated a significant reduction in thymic lymphocytes in the CIAV-challenged control group, accompanied by a blurred boundary between the thymic cortex and medulla and a markedly thinner cortex, which indicated typical ClAV-induced damage. In contrast, the thymus tissue from chickens challenged with ClAV after rMDV immunization showed no such changes and appeared similar to the healthy control group (Figure 6D). Collectively, these results suggested that rMDV vaccination confers robust immune protection against ClAV infection.

**Table 3:** Thymus/body index after CIAV challenge.

Group	1 week Post CIAV challenge
Mock	4.01 ± 0.32
CIAV challenged	2.80 ± 0.39
rMDV-CIAV	4.15 ± 0.29

Different lowercase letters represent statistically significant differences between the groups (*p* < 0.0001).

## 4 Discussion

CIA poses significant challenges to the poultry industry because of its adverse effects on flock health and productivity. The prevalent use of live CIA vaccines is limited to older chickens for safety reasons; however, CIA outbreaks primarily affect young chickens, highlighting the urgent need for early stage immunization strategies. To induce long-lasting immunity from the early stages of a chick’s life, this study utilized the MD vaccine strain rMS-△Meq as a vector to develop a recombinant virus capable of simultaneously expressing CIAV VP1 and VP2 proteins. Our study demonstrated that rMDV not only maintains genomic stability but also consistently expresses CIAV VP1 and VP2 proteins, thereby successfully eliciting both humoral and cell-mediated immune responses. Importantly, the recombinant virus conferred protection against CIAV challenge, addressing the critical need for early-stage immunity in poultry. This new vaccine not only facilitates early induction of active immunity but may also offer passive immunity, though further confirmation is needed.

Construction of MDV recombinant live vector vaccines typically involves several methods, which each present various complexities. The traditional co-transfection of plasmids and viral genomes is complex and time-consuming. Despite its established use in the development of recombinant MDV, the classic BAC method can encounter difficulties in the excision of BAC sequences (Zhao et al., 2008; Zhou et al., 2010; Ma et al., 2014; Liu et al., 2015). In contrast, CRISPR/Cas9 gene editing technology enables specific and efficient DNA modification for gene targeting, thus offering superior speed and efficiency compared to traditional methods (Mei et al., 2016). This advanced technology has been extensively applied for genome manipulation of herpesviruses (Chen et al., 2018), as evidenced by the development of recombinant viruses with targeted modifications, such as herpes simplex virus (HSV) lacking IFI16 (Diner et al., 2016), human Cytomegaloviruses virus (CMV) with a deletion in TSC2 (Bai et al., 2015), Kaposi’s sarcoma-associated herpesviruses (KSHV) without the cell kinase RSK (Avey et al., 2015), Epstein–Barr virus (EBV) expressing a DsRed marker (Yuen et al., 2015), Turkey herpesvirus (HVT) with the NDV fusion protein (Calderón et al., 2022), and MDV co-expressing reporter genes (Li et al., 2022). In this study, we harnessed the power of CRISPR/Cas9, which was combined with eGFP-based flow sorting technology. Compared with other methods of constructing and rescuing a novel recombinant MDV that co-expresses CIAV VP1 and VP2 proteins, our streamlined approach involves the co-transfection of donor and sgRNA plasmids, followed by infection with a specific dose of the parental strain. This approach simplifies the process, accelerates the timeline, and improves editing efficiency by utilizing the HDR (Homology-directed) repair pathway (Tang et al., 2018). Using this approach, the desired rMDV could be successfully rescued within a week. Pure rMDV was obtained after 4–5 rounds of fluorescence flow sorting. The entire experimental cycle can be completed in just 1 month, thereby significantly reducing both the time and difficulty typically associated with rescuing recombinant MDV. This finding underscores the convenience and efficiency of MDV gene manipulation and the construction of MDV-based vector vaccines, and it sets a new standard in the field of vaccine development.

A substantial correlation has been observed between immune protection and antibody levels (Markowski-Grimsrud and Schat, 2003). Currently, most DNA and subunit recombinant CIA vaccines stimulate the production of the relevant antibodies through multiple immunizations. For example, the CIAV recombinant NDV vaccine (Chellappa et al., 2021) and rVP1 and IL-12 recombinant subunit vaccines (Tseng et al., 2019) can only induce the production of specific ELISA antibodies following booster immunization. The antibody titers induced by these vaccines significantly affect their immune efficacy. A combined DNA vaccine and recombinant protein vaccine expressing VP1 and VP2 proteins (Liu et al., 2022) achieved an ELISA antibody titer of approximately 7 × 10^3^ at the fourth week post-booster immunization. Similarly, chickens vaccinated with the DNA vaccine (pBudVP2-VP1/VP22) (Moeini et al., 2011b) experienced a substantial increase in antibody titer to 2.7 × 10^3^ following booster immunization. Compared with these CIA vaccines, a single dose of rMDV immunization in 1-day-old SPF chickens was adequate to induce a high antibody titer (10^5^). Additionally, previous studies have shown that chickens induce neutralizing antibodies only after immunization with recombinant vaccines that co-express VP1 and VP2 proteins (Moeini et al., 2011a). Notably, vaccines expressing only VP1 (Moeini et al., 2011a) or a combination of rVP1 and chIFN-*γ* (Shen et al., 2015) did not produce neutralizing antibodies. In this study, we observed that rMDV co-expressing VP1 and VP2 proteins could effectively stimulate the production of neutralizing antibodies following immunization. In summary, rMDV not only induces high levels of ELISA antibodies but also stimulates the production of neutralizing antibodies. Furthermore, it triggers cellular immunity, making it a promising candidate vaccine strain for the prevention of CIAV.

Anemia and thymic atrophy are the main clinical symptoms observed in chickens infected with CIAV (McNulty, 1991; Todd, 2000; Orakpoghenor, 2019; Fang et al., 2023). In this study, rMDV-vaccinated chickens largely evaded the typical CIAV-induced thymic atrophy and anemia responses, whereas the control group showed severe symptoms, underscoring the protective efficacy of rMDV against CIAV pathologies. Previous studies indicated that the production of specific antibodies against ClAV inhibits viral replication (Otaki et al., 1992; Shulman and Davidson, 2017). The rMDV-vaccinated group showed a significant decrease in viral titer compared to the challenge control group, demonstrating that rMDV immunization effectively suppressed viral replication in the thymus. Based on the antibody levels induced by rMDV, we hypothesized that rMDV inhibits viral replication by inducing antibodies specific to CIAV. In summary, these findings indicated that rMDV effectively reduced the occurrence of anemia and thymic atrophy caused by CIAV infection, thereby providing robust immune protection.

Taken together, we successfully established a convenient and efficient procedure for constructing an rMDV that delivers exogenous immunogenic CIAV proteins. More importantly, this recombinant virus provided sufficient protection against CIA caused by virulent CIAV strains in chickens. Our research provides crucial groundwork and serves as a fundamental platform for the future development of multivalent vaccines against CIA and a broader spectrum of avian infectious diseases, thereby enhancing the arsenal of tools available for poultry health and disease management.

## Data Availability

The raw data supporting the conclusions of this article will be made available by the authors, without undue reservation.
